# Functional and phenotypical comparison of myofibroblasts derived from biopsies and bronchoalveolar lavage in mild asthma and scleroderma

**DOI:** 10.1186/1465-9921-7-11

**Published:** 2006-01-23

**Authors:** Kristoffer Larsen, Johan Malmström, Marie Wildt, Camilla Dahlqvist, Lennart Hansson, György Marko-Varga, Leif Bjermer, Agneta Scheja, Gunilla Westergren-Thorsson

**Affiliations:** 1 Experimental Medical Science, Division of Vascular and Airway Research, Lund University, S-221 84 Lund, Sweden; 2 Institute for Molecular Systems Biology, ETH Hönggerberg, CH-8093 Zürich, Switzerland; 3 Department of Rheumatology, Lund University Hospital, S-221 85 Lund, Sweden; 4 Department of Respiratory Medicine and Allergology, Lund University Hospital, S-221 85 Lund, Sweden; 5 Analytical Chemistry, Lund University, S-221 00 Lund, Sweden

## Abstract

**Background:**

Activated fibroblasts, which have previously been obtained from bronchoalveolar lavage fluid (BALF), are proposed to be important cells in the fibrotic processes of asthma and scleroderma (SSc). We have studied the motility for BALF derived fibroblasts in patients with SSc that may explain the presence of these cells in the airway lumen. Furthermore, we have compared phenotypic alterations in activated fibroblasts from BALF and bronchial biopsies from patients with mild asthma and SSc that may account for the distinct fibrotic responses.

**Methods:**

Fibroblasts were cultured from BALF and bronchial biopsies from patients with mild asthma and SSc. The motility was studied using a cell migration assay. Western Blotting was used to study the expression of alpha-smooth muscle actin (α-SMA), ED-A fibronectin, and serine arginine splicing factor 20 (SRp20). The protein expression pattern was analyzed to reveal potential biomarkers using two-dimensional electrophoresis (2-DE) and sequencing dual matrix-assisted laser desorption ionization time-of-flight mass spectrometry (MALDI-TOF-TOF). The Mann-Whitney method was used to calculate statistical significance.

**Results:**

Increased migration and levels of ED-A fibronectin were observed in BALF fibroblasts from both groups of patients, supported by increased expression of RhoA, Rac1, and the splicing factor SRp20. However, these observations were exclusively accompanied by increased expression of α-SMA in patients with mild asthma. Compared to BALF fibroblasts in mild asthma, fibroblasts in SSc displayed a differential protein expression pattern of cytoskeletal- and scavenger proteins. These identified proteins facilitate cell migration, oxidative stress, and the excessive deposition of extracellular matrix observed in patients with SSc.

**Conclusion:**

This study demonstrates a possible origin for fibroblasts in the airway lumen in patients with SSc and important differences between fibroblast phenotypes in mild asthma and SSc. The findings may explain the distinct fibrotic processes and highlight the motile BALF fibroblast as a potential target cell in these disorders.

## Background

Excessive extracellular matrix (ECM) deposition in skin and internal organs such as the lungs is one of the features of SSc [1]. A similar process occurs in patients with mild asthma where the fibrosis is limited to the peribronchial areas of the lung [2]. Due to the ability of fibroblasts to regulate the normal ECM turnover, these cells are considered to be important in fibrosis [3,4]. Since fibrosis is far more dominant in SSc as opposed to that in mild asthma, one would anticipate finding differences in characterizing the respective fibroblasts. The actual tissue sources of fibroblasts in fibrosis are not completely understood, but the residing fibroblast pool has been suggested to contain different clones that may account for the disease pathology [5]. In addition, the recruitment of circulating fibroblast progenitor cells such as fibrocytes, and the involvement of epithelial mesenchymal transition have been proposed as complementary sources to the residing tissue fibroblast pool in fibrotic disorders [6-9].

In the early phase of airway remodeling, fibroblasts migrate into the tissue, an event that is facilitated by proteins associated with the actin cytoskeleton and intracellular signaling pathways involving small GTPases such as RhoA and Rac1, which induce formation of stress fibers and focal adhesions [10]. Once activated, fibroblasts acquire a myofibroblast phenotype that is characterized by an increased expression of α-SMA and an increased secretion of ECM molecules [4]. This differentiation process can be induced by factors such as transforming growth factor-beta (TGF-β) and alternatively spliced fibronectin that contains the type III extra domain A (ED-A fibronectin) [11,12]. The splicing factor SRp20 has been suggested to be important in determination of site selection on the pre-mRNA in exon inclusion of ED-A fibronectin [11,13].

Activated fibroblasts have previously been cultured from bronchial biopsies from patients with mild asthma and SSc, which has led to new insights into these disorders [14,15]. Furthermore, fibroblasts have been obtained from BALF from patients with SSc, and recently also from patients with mild asthma where increased motility and deposition of ECM components were important features for these cells [16,17]. The BALF fibroblasts are likely to play an important role in the early stages of airway remodeling due to the specific ECM production observed, including increased levels of the pro-fibrotic proteoglycans biglycan and versican. In patients with mild asthma, the increased motility observed in BALF fibroblasts was suggested to account for the presence of these cells in the airway lumen, however, this possible linkage has not been studied in SSc-derived BALF fibroblasts.

In this study, we hypothesized that BALF fibroblasts in SSc display alterations in cell motility which may account for the presence of these cells in the airway lumen. Furthermore, we hypothesized that there are phenotypic distinctions between BALF fibroblasts from patients with mild asthma and SSc, which may account for the different fibrotic processes observed in these disorders. Differences in fibroblast migration, splicing of ECM, and protein expression pattern may reveal new biomarkers and mechanisms involved in the severe disease pathology of SSc.

## Methods

### Subjects, bronchoalveolar lavage, and sampling of lung tissue

Patients suffering from SSc and alveolitis (n = 10, 4 male/6 female) aged 29–69 diagnosed by HRCT were included in the study. All patients met the standards for the American College of Rheumatology criteria for SSc. Four patients had diffuse cutaneous SSc and six had limited cutaneous SSc. The patients were not treated with any putative disease modifying drugs.

Patients with mild asthma with BALF fibroblasts (n = 5, 4 female/1 male) fulfilled the criteria of the American Thoracic Society. These patients had a positive phadiotope staining, PD_20 _< 2 mg/ml of methacholine stimulation, stable asthmatic conditions, free of infections 6 weeks before bronchoscopy, and no corticosteroid treatment 6 months before the study. Informed consent was given from all subjects in the study. A more thoroughly description of these patients with mild asthma have been presented earlier [16]. BAL was performed by flushing the airways with up to 140 ml of 0.9% NaCl, and the recovered fluid was used for analysis. Bronchial biopsies were collected as previously described [14]. This study was fully approved by the local ethical committee (LU 193-01 and LU 339-00).

### Cell cultures

Fibroblasts were cultured from the BALF and bronchial biopsies from patients with mild asthma and SSc as previously described [14]. Fibroblasts were used in passage 5–7. For western blots, the cells were harvested in lysis-buffer containing 10% glycerol, 1% Nonidet 40, 50 mM Tris, 100 mM NaCl, 2 mM MgCl_2_, 2 mM Na orthovanadate, 1 μg/ml PMSF, 1 μg/ml aprotinin and 20 μg/ml leupeptin.

### Western blot

The protein content of the lysed cells was determined using a Bradford protein reagent kit (Pierce, Rockford, IL). Equal amounts of protein were loaded on 4–12% Bis-Tris gels (Invitrogen, Uppsala, Sweden) with MOPS running-buffer. Western Blotting was performed as previously described [18]. The separated proteins were incubated with primary antibodies against human α-SMA (DAKO, Glostrup, Denmark), human RhoA (Santa Cruz Biotech, Santa Cruz, CA), human Rac1 (Transduction Labs, Lexington, KY), human ED-A/Fibronectin (Abcam Ltd, Cambridge, Cambridgeshire, UK), human TGF-β (R&D Systems, Abingdon, UK), or human SRp20 (Zymed Labs Inc, South San Francisco, CA). A secondary HRP-labelled rabbit-antimouse (DAKO, Glostrup, Denmark) antibody was used and the intensity of the bands on the membrane were analysed using the Gel-Pro™ Analyser software (Media Cybernetics, Silver Spring, MD).

### Cell migration assay

The migration of the cultured fibroblasts was analyzed as previously described [18]. Briefly, fibroblasts (30,000 cells) were cultured for 6 hours within a cloning cylinder. The cylinder was removed and the fibroblasts were allowed to migrate for 48 h. The cells were fixed in 1% glutaraldehyde and stained for 2 hours in 0.5% crystal violet prior to the distance measurements. The migration was measured as distance traveled for 200 cells from the border of the removed cylinder.

### Stress fiber analysis

For stress fiber analysis, cells were seeded (5000 cells/well) under the conditions described above. Thereafter, cells were fixed in 4% paraformaldehyde in PBS for 15 minutes. After permeabilization in 0.5% Triton X-100 in PBS for 5 minutes, and blocking with 1% BSA in PBS for 30 minutes, the cells were incubated for 30 minutes with Alexa Fluor™ 488 phalloidin probe (Molecular Probes, The Netherlands) diluted in blocking buffer. Cells were rinsed carefully between each step. A Nikon Microphot-FXA fluorescent microscope (Nikon, Japan) was used to study the cells. Monoclonal mouse antibody against paxillin was used, followed by Alexa Fluor™ 584 goat-anti-mouse IgG (Molecular Probes, The Netherlands).

### Proteome expression

Two-dimensional 2-DE was performed as previously described [16]. Briefly, cells were harvested in solubilization solution (7 M urea, 2 M thiourea, 2% ((chloamidopropyl)-dimethylammonio)- propanesulfonate (CHAPS). 10 mM dithiotreitol (DTT) and 0.33% immobilized pH gradient (4–7) buffer (IPG) (Amersham Biosciences, Uppsala, Sweden) were added to the samples, which were rehydrated with Immobiline DryStrips (180 mm, pH 4–7, Amersham Biosciences, Uppsala, Sweden). The isoelectric focusing step was performed using a Multiphor^® ^II (Amersham Biosciences, Uppsala, Sweden) according to the following schedule: 300 V 1 min, 3500 V 25 h until approximately 85 kVh were reached. The strips were applied on 14% homogeneous duracryl gels and electrophoresis was performed at 100 V for 18 h using a Hoefer™ DALT gel apparatus (Amersham, San Francisco, CA).

Gels were stained by silvernitrate according to Shevchenko et al. [19] and scanned using a Bio-Rad GS-710 gel scanner (Bio-Rad, Hercules, CA). Preparative gels were stained using Brilliant Blue G-Colloidal (Sigma-Aldrich, Saint-Louis, MO) according to the instructions from the manufacturer. Image analysis was performed using PDQuest 7.01 2-D gel analysis software (Bio-Rad, Hercules, CA). Each spot on the gel was given an integrated optical density (IOD) value by the software that was compared to the total amounts of spots and is therefore referred to as ppm of the total IOD of all valid spots. The statistical evaluation of the differential protein expression pattern was performed using Ludesi Interpreter software (Ludesi AB, Lund, Sweden). Protein spots that displayed a two-fold or larger differential expression pattern were considered as spots of interest. These spots were excised from the gels, washed with 50 mM ammonium bicarbonate buffer, followed by three rounds of acetonitrile, treated overnight with 10 ng/ml trypsin (Promega, Madison WI) and acidified with 0.5% trifluoric acid.

The samples were desalted and concentrated by Ziptip (Millipore, Bedford, MA) according to the manufacturer's instructions and thereafter placed on polished stainless steel target plates together with 7.5 mg/mL a-cyano-4-hydroxycinnamic acid dissolved in 60:40 acetonitrile-water. The MALDI plates were analyzed in automated mode on the AB4700 Proteomics Analyzer (Applied Biosystems, Framingham, MA) with 1000 laser shots in MS mode and with internal two-point calibration on trypsin peptides. MS/MS spectra were acquired using up to 3000 laser shots/precursor unless the pre-defined signal-to-noise level in the MS/MS acquisition was achieved sooner. The MS/MS data were submitted for database to Mascot (Matrix Science Inc. Boston, MA) with a parent mass error tolerance of 25 ppm and mass fragments with an error tolerance of 0.2 Da.

### Statistical methods

Mean values ± standard error of the mean (SEM) were calculated and the Mann-Whitney method was used for analyses of statistical significance. All values of p < 0.05 (*) were considered significant.

## Results

### BALF fibroblasts in SSc display increased migration and expression of small GTPases

**Figure 1:**
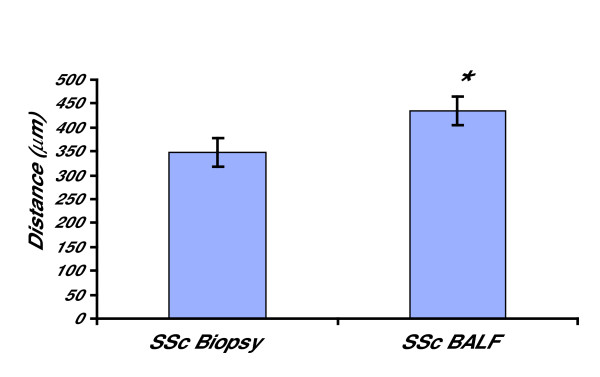
**Migration of fibroblasts from BALF and bronchial biopsies from patients with SSc**. Fibroblasts were cultured from BALF and bronchial biopsies in a "clone-cylinder", which was removed after 24 h. The distance from the border of the removed cylinder covered by the cells was measured after an additional 48 h. Values are presented as means ± SEM for n = 5 patients/group. *Significant difference when comparing the migration between BALF fibroblasts and biopsy fibroblasts from patients with SSc.

BALF fibroblasts were established from 5 out of 10 patients with SSc. This is similar to previous findings in patients with mild asthma where these cells could be established from 5 out of 12 patients [16]. To study if BALF fibroblasts in SSc could originate from the submucosa, differences in cell migration were studied in fibroblasts from BALF and bronchial biopsies from patients with SSc. A significant 1.2-fold (p < 0.05) increase in cell migration was reported for the BALF fibroblasts from patients with SSc when compared to fibroblasts from bronchial biopsies (Fig 1). These observations are in accordance with previous findings where BALF fibroblasts from patients with mild asthma displayed a significant increase in cell migration [16].

**Figure 2:**
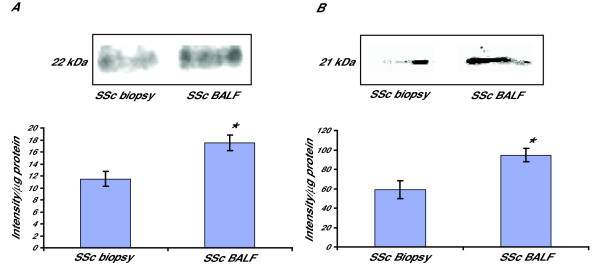
**Expression of RhoA and Rac1 in fibroblasts from BALF and bronchial biopsies from patients with SSc and mild asthma**. Fibroblasts were harvested in lysis buffer as described in the Method section. Equal amounts of protein were loaded on 4–12% Bis-Tris gels. Western Blotting was performed to study the expression of RhoA (A) and Rac1 (B) where the optical density of the bands was measured to determine the expression. Values are presented as means ± SEM for n = 5 patients/group. *Significant difference when comparing the expression of RhoA and Rac1 between BALF fibroblasts and biopsy fibroblasts from patients withSSc.

To study if the increased migration of BALF fibroblasts was linked to the expression of the small GTPases RhoA and Rac1, Western Blotting was performed on cultured fibroblasts. Several studies have demonstrated that these proteins are of importance in cell migration [10]. A significant increase of RhoA (1.5-fold increase, p < 0.05) and Rac1 (1.3-fold increase, p < 0.05) was observed in the BALF fibroblast cultures (Figs 2A–B). These increases are also in accordance with previous results from BALF fibroblasts from patients with mild asthma [16].

### Increased levels of ED-A fibronectin and SRp20 in BALF fibroblasts

**Figure 3:**
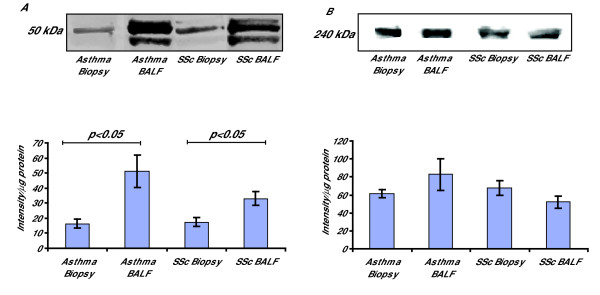
**Expression of fibronectin isoforms in fibroblasts from BALF and bronchial biopsies from patients with mild asthma and SSc**. Fibroblasts were cultured from bronchial biopsies and BALF from patients with SSc and mild asthma and harvested in lysis buffer as described in the Method section. Equal amounts of protein were loaded on 4–12% Bis-Tris gels. The production of the alternatively spliced isoform ED-A fibronectin (A) and cellular fibronectin (B) was measured using Western Blot with human ED-A fibronectin and cellular fibronectin antibodies. Quantification of fibronectin expression was performed by measuring the optical density of the bands. Values are presented as means ± SEM for n = 5 patients/group.

Next we examined the production of the alternatively spliced form of cellular fibronectin, ED-A fibronectin, in BALF fibroblasts, which was compared to that of fibroblasts from bronchial biopsies in patients with SSc and mild asthma. A significant 2-fold increase (p < 0.05) was seen in the BALF fibroblast cultures from patients with SSc and a 5-fold increase (p < 0.05) was observed in BALF fibroblasts from patients with mild asthma when compared to biopsy fibroblasts (Fig 3A). The antibody used reacts with an epitope located in the ED-A sequence of cellular fibronectin. Analysis of the immunological determinant recognized by the antibody shows three fragments of 47, 44, and 52 kDa. Interestingly, the three fragments were visible on the Western Blot membrane in BALF fibroblasts from both disorders, but only one fragment was seen in the biopsy cultures from patients with mild asthma and SSc. No difference in total production of fibronectin was seen between the two fibroblast phenotypes in patients with SSc or mild asthma (Fig 3B). The proportion of ED-A fibronectin in patients with SSc and asthma from the BALF fibroblasts was 60% ED-A, whereas in the biopsy cultures 25% of the total fibronectin production was ED-A fibronectin.

**Figure 4:**
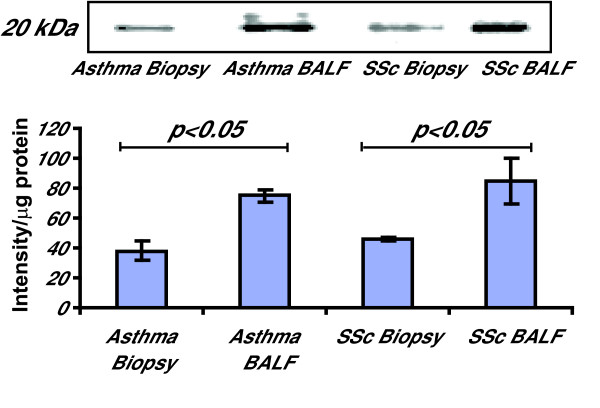
**Production of SRp20 in fibroblasts from BALF and bronchial biopsies from patients with SSc and mild asthma**. Fibroblasts were harvested in lysis buffer as described in the Method section. Equal amounts of protein were loaded on 4–12% Bis-Tris gels. The level of SRp20 expression was determined by Western Blotting and further quantified by measuring the optical density of the bands. Values are presented as means ± SEM for n = 5 patients/group.

Next, we studied if the serine-arginine (SR) splicing factor SRp20 was regulated, since it has been proposed to be involved in the increased expression of alternative splicing of fibronectin. A 1.4-fold increase of SRp20 expression was seen in BALF fibroblasts from patients with asthma (p < 0.05) and a 1.3-fold increase was observed in SSc when compared to fibroblasts from bronchial biopsies (p < 0.05) (Fig 4).

### Cultured fibroblasts from BALF and biopsies in mild asthma and SSc display myofibroblast phenotype and unaltered TGF-β production

**Figure 5:**
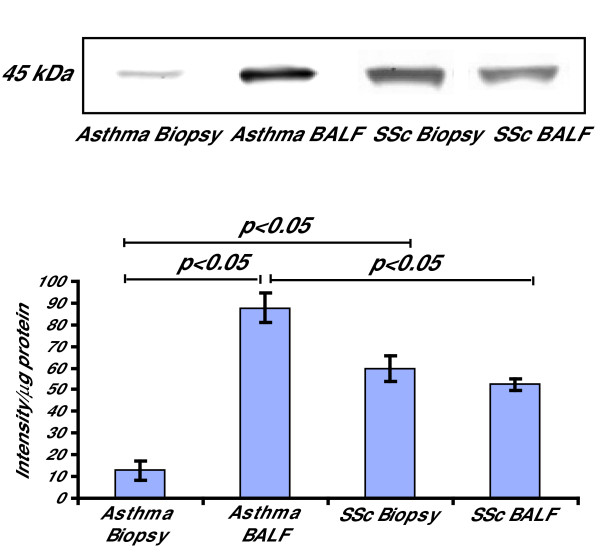
**Expression of α-SMA in fibroblasts from BALF and bronchial biopsies from patients with SSc and mild asthma**. Fibroblasts were cultured from bronchial biopsies and BALF from patients with SSc and mild asthma and harvested in lysis buffer as described in the Method section. Equal amounts of protein were loaded on 4–12% Bis-Tris gels. The expression of α-SMA was detected using Western Blot with human α-SMA antibodies and further quantified by measuring the optical density of the bands. Values are presented as means ± SEM for n = 5 patients/group.

**Figure 6:**
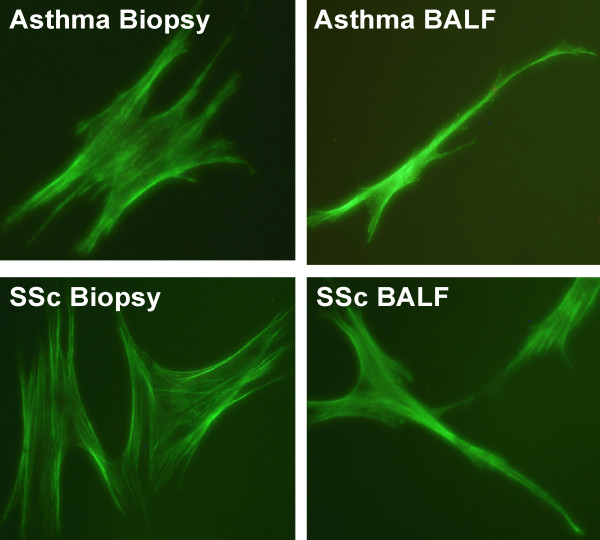
**Actin expression in fibroblasts from BALF and bronchial biopsies from patients with SSc and mild asthma are arranged into stress fibers**. Fibroblasts were cultured from bronchial biopsies and BALF from patients with SSc and mild asthma. Cells were seeded on four-well chamber slides (5000 cells/well), stained with Alexa Fluor™ 488 phalloidin showing stress fibers and analyzed using a fluorescence microscope.

It has been shown that increased ED-A fibronectin levels are associated with increased α-SMA levels [12]. Therefore, the cells were analyzed for expression of the myofibroblast marker α-SMA to study the phenotype of the cultured fibroblasts. The fibroblasts expressed α-SMA in both groups of patients where a significant 8-fold (p < 0.05) increase was observed in BALF fibroblasts from patients with mild asthma when compared to fibroblasts from bronchial biopsies (Fig 5A). When compared to BALF fibroblasts in SSc, the BALF fibroblasts from patients with mild asthma displayed a 1.3-fold increase in α-SMA expression (p < 0.05). No difference in α-SMA expression was observed between the two groups of SSc fibroblasts, but the expression was larger (5-fold, p < 0.05) in SSc biopsy fibroblasts than in biopsy fibroblasts from patients with mild asthma. The actin filaments in all cells were arranged into stress fibers (Fig 6). These findings suggest similar phenotypes in BALF- and biopsy fibroblasts in SSc but a larger difference in α-SMA expression in BALF fibroblasts from patients with mild asthma when compared to corresponding biopsy fibroblasts.

The production of TGF-β, which has been shown to induce a myofibroblast phenotype with elevated levels of α-SMA expression in cultured fibroblast, was studied in the fibroblasts from BALF and bronchial biopsies in both patient groups. A tendency towards reduced TGF-β production was seen for the BALF fibroblast cultures from patients with SSc when compared to asthma, however, this decrease was not statistically significant (data not shown). No alterations were seen in production between the remaining cultured BALF- and biopsy fibroblasts in patients with SSc or mild asthma (data not shown).

### BALF fibroblasts from patients with SSc and mild asthma display a differential proteome

**Figure 7:**
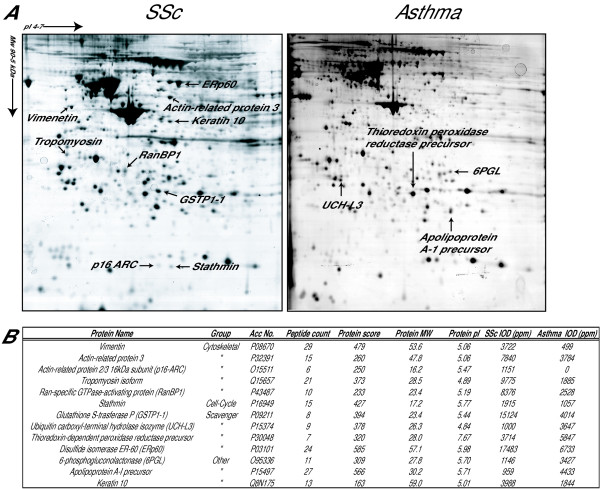
**Protein expression pattern in BALF fibroblast cultures from patients with SSc and mild asthma**. Cells were cultured in six-well plates and harvested as described in the Method section. The lysed cells were separated by 2-DE. A representative 2-D gel from asthma and SSc BALF fibroblasts are presented and the significant differentially expressed spots when are marked with arrows (A). The identified differentially expressed proteins in fibroblasts from BALF in patients with mild asthma and SSc was identified using sequencing MALDI-TOF-TOF (B). IOD (ppm) is the optical density of the spots correlated to the total optical density for all spots present in the gel. A*bbreviations*: Acc No. = Swissprot accession number; MW = Molecular weight. IOD (ppm) = Optical density of the spots correlated to the total optical density for all spots present in the gel. Peptide count = Number of identified peptides that could be matched to the suggested database protein. Protein Score = Probability that the peptide counts are derived from the suggested database protein.

The next set of experiments was addressed to explore differences between SSc and mild asthma on a molecular level by using two-dimensional gel electrophoresis (2-DE) in the range of pH 4–7 and MALDI-TOF-TOF, in the hope of revealing important markers for the different fibrotic processes. A series of triplicate gels were studied where the master gel for each patient group comprised of approximately 500 unique protein spots. The differential protein expression pattern between BALF fibroblasts from patients with SSc and mild asthma displayed 24 differentially expressed spots of statistical significance (p < 0.05). Of these differentially expressed spots, 13 protein spots displayed a statistical significant 2-fold or larger difference in expression and these were matched in all gels (Fig 7A). A protein score >57 with more than two matched peptides were considered to be a significant identification. We were able to identify 6–29 peptides that yielded a protein score ranging from 163–585, thus indicating a high probability for the identified proteins. These proteins were divided into different groups depending on their functional role (Fig 7B); cytoskeletal-associated, cell cycle regulating-, scavenger- and metabolic proteins. The proteins that displayed the largest differences in protein expression were cytoskeletal associated proteins and scavenger proteins.

The proteome in fibroblasts from BALF and bronchial biopsies from patients with mild asthma have been previously shown to include differentially expressed proteins involved in cell migration [16]. However, when comparing the fibroblast proteome between BALF and bronchial biopsies from patients with SSc, only three proteins displayed a significant differential expression pattern (data not shown). Again, this indicates that BALF- and biopsy fibroblast cultures of patients with SSc are more similar in phenotype, which correlates to the similar levels of α-SMA expression (Fig 5). In addition, a comparison between asthma biopsy fibroblasts and SSc biopsy fibroblasts were performed, however no significant regulated proteins could be observed using this approach.

## Discussion

In this study, we have reported increased motility in BALF fibroblasts from patients with SSc when compared to fibroblasts from corresponding bronchial biopsies, which proposes a possible mesenchymal origin for these cells. The increased migration is in accordance with previous studies on BALF fibroblasts from patients with mild asthma [16]. This finding was accompanied by an elevated expression of the small GTPases RhoA and Rac1. These observations are important since RhoA and Rac1 have been suggested to be involved in cell migration by formation of stress fibers as well as the formation and maintenance of focal adhesions [20]. Differences in phenotype between fibroblasts cultured from bronchial biopsies and BALF from patients with SSc and mild asthma were characterized which may be key factors in the distinct fibrotic responses of these disorders. The production of ED-A fibronectin was elevated in BALF fibroblasts from patients with SSc and mild asthma when compared to fibroblasts cultured from corresponding bronchial biopsies. This alternatively spliced form of cellular fibronectin that contains the ED-A domain is associated with wound healing and fibrosis in diseases such as SSc [11]. The expression of this matrix molecule serves as a marker of extracellular matrix that is closely linked to intracellular α-SMA expression myofibroblasts [21]. Furthermore, the elevated levels of ED-A fibronectin in BALF fibroblasts may also explain the increased migration observed in these cells [22]. The elevated levels of the splicing factor SRp20 in the BALF fibroblast cultures may explain the induced expression of ED-A fibronectin. TGF-β specifically induces the expression of SRp20, which when over-expressed promotes the alternative splicing of fibronectin [13]. The TGF-β-induced expression of ED-A fibronectin is required for TGF-β-triggered increase of α-SMA [12]. The cultured fibroblasts from BALF and bronchial biopsies from SSc and mild asthma expressed α-SMA, which suggests that these cells display a myofibroblast phenotype. The increased expression of α-SMA in BALF fibroblasts from patients with mild asthma and SSc proposes distinct BALF fibroblast phenotypes in these disorders. Although the levels of α-SMA expression in fibroblasts from BALF and bronchial biopsies in SSc did not display a distinct pattern, the levels were elevated when compared to biopsy fibroblast in mild asthma. These observations are important since they may reflect the differentiated stage the cells are cultured from, which in turn may reflect the degree of fibrosis in the tissue.

Since BALF fibroblasts in SSc and mild asthma display increased migration and express important myofibroblast markers such as α-SMA and ED-A fibronectin, the differential protein expression profile between the two BALF fibroblast groups were studied by using 2-DE and MALDI-TOF-TOF to reveal factors that may account for the distinct fibrotic processes in these disorders. This approach is an excellent tool when identifying high abundant proteins but less efficient when studying membrane associated- and low abundant proteins. Nevertheless, many of the high abundant proteins within range for the 2-DE are involved in important cellular mechanisms, including fibrosis. Cytoskeletal proteins, such as vimentin, tropomyosin, and actin associated proteins were identified in elevated levels in the SSc BALF fibroblasts, which may all account for the motile phenotype that characterizes the BALF fibroblast. Moreover, these proteins are elevated in activated myofibroblasts since they have been suggested to be involved in the increased intracellular trafficking and secretion of ECM molecules, which is an important feature of the myofibroblast in fibrotic tissue. Ran-binding protein 1 (RanBP1) has been shown to be induce migration [23]. This protein was expressed in BALF fibroblasts from patients with mild asthma and SSc, but was significantly increased in the latter group. This observation may therefore explain the increased migration characteristic for the BALF fibroblasts from patients with SSc. Several scavenger proteins involved in oxidative stress and redox processes such as disulfide isomerase (ERp60) and glutathione S-transferase P (GSTP1-1), displayed elevated levels in BALF fibroblasts from patients with SSc. Interestingly, oxidative stress is considered an important factor in patients with SSc in contributing to vascular damage leading to an activation of fibroblasts and inflammatory cells [24]. Therefore, the increased levels of scavenger proteins in BALF fibroblasts from patients with SSc may reflect a response to the elevated levels of oxidative stress, mediated by free radicals observed in patients with SSc.

The small number of differentially expressed proteins between fibroblasts from BALF and bronchial biopsies from patients with SSc suggests that these two fibroblast phenotypes are relatively similar, an observation that was further supported by the small differences in α-SMA expression. In contrast to this observation, BALF fibroblasts from SSc and mild asthma display important distinctions in α-SMA and protein expression pattern. These observations emphasize the complex diversity of myofibroblast phenotypes present in the human fibrotic lung, which have been shown in previous studies to exhibit different affinity and activation from cytokines and growth factors such as TGF-β [25]. Fibroblasts have the ability to produce TGF-β by themselves through an autocrine mechanism that has been suggested to be of importance in maintaining the myofibroblast phenotype by inducing increased levels of α-SMA [26]. We did not observe, however, any differences in the production of TGF-β from the fibroblasts alone in this study. ECM components such as heparin, biglycan and decorin that are produced by myofibroblasts can affect the differentiation process in an autocrine manner [5,27] and may thus represent a possible TGF-β independent pathway for the observed differences in α-SMA expression. In addition, a contribution of other TGF-β-producing cells in the early passages such as eosinophils and macrophages may also affect levels of ED-A fibronectin reported in the analyzed BALF fibroblasts in later passages [28]. Increased levels of BALF eosinophils have been reported in patients with mild asthma with BALF fibroblasts when compared to patients with asthma and control subjects without the presence of these cells, however if this linkage is present in SSc remains to be elucidated in future studies [16]. The origin of the BALF fibroblasts is not known but since fibroblasts reside in areas beneath the basement membrane, it is tempting to speculate that fibroblasts with increased motility would migrate to the airway lumen upon possible stimuli or damages to the airway epithelium. Another possible origin for the BALF fibroblasts includes the recruitment of fibroblast progenitor cells, termed fibrocytes, from the circulation. In SSc, the endothelial cells are damaged by mediators such as free radicals, which may facilitate trafficking of cells from the circulation through the endothelium to interact with fibroblasts [24].

## Conclusion

The characterization of BALF fibroblasts from patients with SSc and the comparison with patients with mild asthma emphasize the importance of activated fibroblasts in these disorders. The increased motility in fibroblasts derived from BALF when compared with those derived from bronchial biopsies suggests a potential submucosal origin for these cells. Moreover, the findings in this study highlight important distinctions in fibroblast phenotype between the two disorders which may reflect the different disease pathology in SSc. This makes the BALF fibroblast an interesting target cell for future therapies of lung fibrosis observed in SSc.

## Competing interests

The author(s) declare that they have no competing interests.

## Authors' contributions

KL: drafted the manuscript, participated in the organization of the manuscript, performed all two-dimensional 2-DE experiments and sample preparation prior to the mass MALDI-TOF-TOF analysis, and performed some of the cell migration assays and cell culture experiments.

JM: performed all mass MALDI-TOF-TOF analyses and peptide database searches. He also participated in the organization of the manuscript.

MW: handled a majority of the cell culture experiments, as well as participated in the planning of the manuscript.

CD: Technician who performed many of the Western Blot experiments and was also involved in the organization of the manuscript.

LH: Clinician who performed many of the bronchoscopy sessions when collecting SSc biopsies and BALF and was involved in organization of the manuscript.

GMV: One of the initiators of this study who has collaborated in the organization of the manuscript.

AS: One of the initiators of this study who has collaborated in the organization of the manuscript.

LB: Clinician who performed many of the bronchoscopy sessions when collecting asthma biopsies and BALF and was involved in organization of the manuscript.

GWT: Group leader who (in collaboration with AS) initiated the study and organized the manuscript.
